# Interleukin-1β receptor expressed by modified vaccinia virus Ankara interferes with interleukin-1β activity produced in various virus-infected antigen-presenting cells

**DOI:** 10.1186/1743-422X-10-34

**Published:** 2013-01-28

**Authors:** Stefan Zimmerling, Zoe Waibler, Theresa Resch, Gerd Sutter, Astrid Schwantes

**Affiliations:** 1 Division of Virology, Paul-Ehrlich Institute, Langen, Germany; 2 Junior Research Group, Novel Vaccination Strategies and Early Immune Responses, Paul-Ehrlich Institute, Langen, Germany; 3 President´s Research Group 2, Paul-Ehrlich Institute, Langen, Germany; 4 Institute for Infectious Diseases and Zoonoses, LMU University of Munich, Munich, Germany

**Keywords:** Modified vaccinia virus Ankara, Interleukin-1β induction, Viral interleukin-1β receptor, Inflammasome, Caspase-1, Human cells, Macrophages, Dendritic cells

## Abstract

**Background:**

Modified vaccinia virus Ankara (MVA) is a highly attenuated virus and a promising vaccine vector with potent immune stimulating properties. Deletion of the gene encoding the viral interleukin-1beta receptor (vIL-1βR) in MVA (MVAΔIL-1βR) was previously shown to enhance memory T cell function. Here, we investigated the influence of vIL-1βR on blocking interleukin-1beta (IL-1β) upon MVA infection in various antigen presenting cells of murine and human origin, and analyzed whether inflammasome function contributes to IL-1β production in different cell types.

**Findings:**

Extending previous studies, immunizing mice with low doses of MVAΔIL-1βR still showed enhanced memory CD8^+^ T cell activation compared to MVA wild-type (MVAwt) immunization. *In vitro*, murine myeloid dendritic cells, and activated, but not naive primary macrophages were identified as potent producers of IL-1β upon infection with MVA. Importantly, free IL-1β was only detected in the absence of vIL-1βR. Moreover, MVAΔIL-1βR increased amounts of bioactive IL-1β compared to MVAwt after infection of human THP-1 cells, as detected using a reporter system that only responds to active and free IL-1β. The MVA-mediated induction of IL-1β was confirmed to depend on inflammasome function in human and murine cells, however in murine cells this apparently involves caspase-1-independent pathways.

**Conclusions:**

MVA lacking IL-1β blocking activity leads to increased concentrations of free IL-1β upon infection of murine and human antigen presenting cells; this is likely responsible for enhanced memory T cell activation upon MVAΔIL-1βR immunization of mice. Moreover, our results suggest that MVA-mediated IL-1β induction is a multifactorial process.

## Findings

### Background

Modified vaccinia virus Ankara (MVA) is a highly attenuated vaccinia virus (VACV) generated by tissue culture passaging of VACV Ankara, a first generation smallpox vaccine [1]. During this process many viral genes acquired mutations or were deleted, resulting in the loss of productive viral replication in most mammalian cell types [2,3]. This enabled recombinant MVA to be established as efficient and particularly safe VACV vector [4].

In contrast to conventional VACV, MVA is able to potently stimulate innate immunity [5-7] including induction of IL-1β [8]. Paradoxically, the gene encoding the viral interleukin-1beta receptor homolog (vIL-1βR) has remained functional in MVA [3,7]. Viral IL-1βR is expressed late during the VACV life cycle as a secreted protein that binds specifically and with high affinity to mature IL-1β [7,9,10]. The protein has been shown to play a role in the pathogenesis of poxvirus-mediated disease [11], pointing to the importance of blocking the activity of the pro-inflammatory cytokine interleukin-1beta (IL-1β). Deleting the gene encoding vIL-1βR in MVA vaccines has resulted in enhanced memory CD8^+^ T cell responses against MVA or recombinant antigens upon immunization of mice [12-14].

Here, we show the ability of MVA to induce IL-1β in various antigen presenting cells, and indicate the possibility of a different requirement of caspase-1-containing inflammasomes for IL-1β induction. Particularly, we demonstrate that vIL-1βR substantially affects the availability and activity of free IL-1β.

### MVAΔIL-1βR enhances memory CD8^+^ T cell responses in low dose prime-boost immunizations

To explore in more detail the potency of MVA-induced IL-1β on memory T cell activation *in vivo*, we immunized C57BL/6 mice with different low doses of wild-type MVA (MVAwt) or mutant MVA lacking vIL-1βR expression (MAVΔIL-1βR) [12]. Viruses were administered intravenously to elicit a predominantly systemic T cell response that could be measured in secondary lymphatic organs. Ten weeks after primary vaccination, all mice received a booster immunization. Since our previous results in the BALB/c model indicated that priming is critical for differences in memory T cell activation [12], we used MVAwt for the second immunization to provide identical conditions for secondary T cell expansion in all mice.

**Figure 1:**
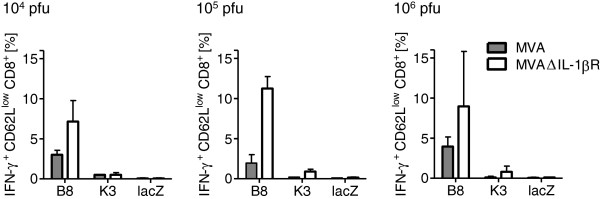
**Memory CD8**^**+ **^**T cell responses in mice is enhanced by low dose MVAΔIL-1βR immunization.** C57BL/6 mice (6–8 week-old; n = 2/group) were immunized intravenously with MVAwt or MVAΔIL-1βR. Mouse groups received increasing doses of 10^4^, 10^5^ or 10^6^ pfu of each virus. Ten weeks after immunization, all mice were boosted intravenously with 10^6^ pfu of MVAwt. Five days later, mice were sacrificed and splenocytes were stimulated with VACV-specific B8R_20-27_ (B8) or K3L_6-15_ (K3) peptides or β-galactosidase_876_ (lacZ) peptide as a control. Three independent *in vitro* stimulations were performed per mouse. Activated (CD62L^low^) IFN-γ producing CD8^+^ T cell populations were quantified by FACS for each immunization dose. The mean of the mice per group is shown with standard deviations.

In agreement with our previous findings [12], we consistently found higher numbers of IFN-γ-secreting activated memory CD8^+^ T cells specific for two different VACV-specific epitopes (K^b^-restricted immunodominant B8R_20-27_; D^b^-restricted subdominant determinant K3L_6-15_[15]) in mice immunized with MVAΔIL-1βR than in those immunized with MVAwt (Figure 1). These new data suggest that even low amounts of MVA induce IL-1β *in vivo*, and that in the absence of vIL-1βR this is sufficient to enhance memory T cell stimulation.

### MVA induces IL-1β in different murine antigen presenting cells but the viral IL-1β receptor effectively blocks this cytokine

We previously hypothesized that MVAΔIL-1βR’s inability to neutralize IL-1β upon infection may lead to improved functionality of DCs, and thus better T cell memory responses [12]. Myeloid dendritic cells (mDCs) are potent antigen presenting cells and important for T cell priming; we therefore investigated their ability to respond to MVA infection by producing IL-1β. We also compared their response to identically treated bone marrow-derived macrophages (BMDMs), which have already been shown to produce IL-1β upon MVA infection [8].

**Figure 2:**
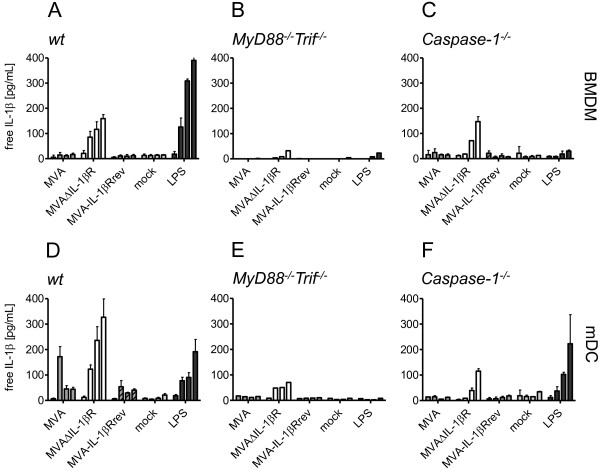
**MyD88, Trif and Caspase-1 are involved in MVAΔIL-1βR-mediated IL-1β induction in murine antigen presenting cells.** Bone marrow-derived macrophages (BMDM) (**A** - **C**) or myeloid dendritic cells (mDC) (**D** - **F**) were generated from C57BL/6 mice (**A**, **D**), MyD88^−/−^Trif^−/−^ mice (**B**, **E**), or Caspase-1^−/−^ mice (**C**, **F**). Cells were differentiated *in vitro* from a pool of isolated bone marrow cells derived from two mice per mouse strain. Cells were infected with MVAwt, MVAΔIL-1βR, or MVA-IL-1βRrev at five MOI, mock treated with medium, or incubated in the presence of 1 μg/ml LPS. Infections with each virus were performed once (**B**, and **E**) or in duplicate (**A**, **C**, **D**, and **F**). At 0, 4, 8 and 24 h p.i. cell free supernatants were subjected to IL-1β ELISA. Since vIL-1βR interferes with IL-1β binding by one ELISA antibody, only unbound, free cytokine is measured in this assay. Levels of free IL-1β are shown for each virus from the shortest to the longest time point (left to right). The mean of infection experiments with standard deviations are shown for **A**, **C**, **D**, and **F.**

To investigate the blocking effect of vIL-1βR on IL-1β induction we compared MVAΔIL-1βR with MVAwt. IL-1β production was detected by ELISA where vIL-1βR interferes with one of the ELISA antibodies binding to IL-1β so only unbound IL-1β is detected. Upon infection of BMDM and mDC with MVAΔIL-1βR, IL-1β production was observed in both cell types, but interestingly, higher IL-1β levels were detected in mDCs. IL-1β was detected 4 hours after infection, and was still increasing up to 24 hours after infection (Figure 2A,D). However, the cytokine was not measurable at late time points upon infection with MVAwt or MVA-IL-1βRrev, a revertant virus with a re-inserted vIL-1βR gene [12], indicating that vIL-1βR has potent blocking activity (Figure 2A,D). Unlike our results, Delaloye et al. observed IL-1β production in BMDM even with MVAwt [8]. However, they used LPS-primed cells, suggesting that activating antigen presenting cells in this manner enhances MVA-mediated IL-1β production. In contrast, our results show that free IL-1β can be produced from unstimulated cells, but only with MVA lacking IL-1β binding capacity.

### MVA-mediated IL-1β production in murine antigen presenting cells involves caspase-1-dependent and -independent mechanisms

MVA is thought to induce IL-1β via the inflammasome in murine and human macrophages [8]. Here, we also wanted to investigate relevant pathways leading to IL-1β activation in murine mDCs. First, we studied the impact of interleukin-1 receptor I and Toll-like receptor signaling on IL-1β activation in mDCs and BMDMs, both generated from MyD88^−/−^Trif^−/−^ knockout mice. Upon infecting both cell types with MVAΔIL-1βR, considerably less free IL-1β was detected than in wt cells (Figure 2B,E vs. 2A,D), suggesting one or both of these molecules are required for IL-1β induction.

To investigate the inflammasome contribution, we focused on caspase-1, a central molecule of several inflammasomes because it cleaves pro-IL-1β into its mature and secreted form [16]. Interestingly, IL-1β was produced in BMDMs and mDCs in the absence of caspase-1. However, levels of free IL-1β were less in mDCs than in wt cells (Figure 2F vs. 2D), but not in BMDMs (Figure 2C vs. 2A). In the latter cell type, IL-1β activation was completely abrogated upon LPS stimulation, confirming the absence of caspase-1 activity (Figure 2C).

These results suggest an alternative caspase-1-independent mechanism of MVA-mediated IL-1β activation in murine antigen presenting cells. Some reports describe that other proteinases such as the neutrophil- and macrophage-derived serine protease proteinase-3, elastases, cathepsin-G and certain matrix metalloproteases also process pro-IL-1β into a secretable cytokine [17-19]. Thus, MVA or poxviruses in general are likely able to activate IL-1β by caspase-1-dependent and independent inflammasomes.

### MVA induces IL-1β in activated primary macrophages

**Figure 3:**
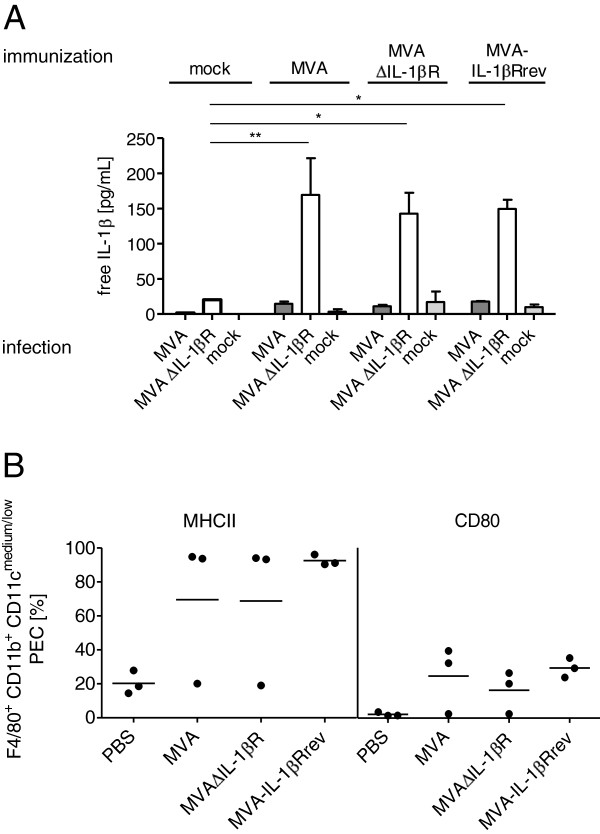
**MVAΔIL-1βR induces IL-1β production in activated mouse peritoneal macrophages.** C57BL/6 mice were immunized intraperitoneally with 10^8^ pfu MVAwt, MVAΔIL-1βR, or MVA-IL-1βRrev, or mock-treated with PBS. Six days after infection, peritoneal exudate cells (PEC) were collected by washing the abdominal cavity with 5–7 ml of PBS. **A** Isolated PEC from each immunized group of mice (n = 2) were pooled and cultured in medium. One day after cultivation, adherent cells (macrophages) were infected in duplicate with MVAwt or MVAΔIL-1βR at five MOI, or mock infected for 24 hours. Means of the two infections per virus and immunization group are shown with standard deviations. Data were evaluated by a mixed linear model for repeated procedures (mouse) with fixed factor immunization group (mock, MVAwt, MVAΔIL-1βR, and MVA-IL-1βRrev). Statistical analyses were performed with SAS®/STAT software, version 9.3, SAS System for Windows. (* = p-value < 0.05 and ** = p-value < 0.01). **B** Three mice/group were immunized with the different viruses as described above. Isolated PEC from each mouse were stained for F4/80, CD11b, and CD11c as macrophage lineage markers and for CD80 and MHC class II as activation markers. Percentages of MHC class II positive (left panel) or CD80 positive (right panel) F4/80^+^, CD11b^+^, and CD11c^medium/low^ PEC from each mouse are represented by one dot. Means of the three mice per group are shown.

We subsequently analyzed IL-1β induction in primary macrophages isolated from the peritoneal cavity of mice. In contrast to the results found for BMDM in Figure 2, infection of these cells with MVAΔIL-1βR did not induce detectable levels of free IL-1β (Figure 3A, immunization: mock). However, when macrophages were isolated from mice immunized with MVAwt or MVA derivatives six days before, *in vitro* infection of these cells with MVAΔIL-1βR again resulted in IL-1β production, regardless of the immunizing virus used (Figure 3A, immunization: MVA variants).

Assuming that peritoneal immunization leads to activation of local macrophages, we investigated levels of activation markers on peritoneal macrophages after immunization with MVA viruses. Indeed expression levels of MHC class II and CD80 were elevated (Figure 3B). Interestingly, when we investigated IL-1β induction *in vivo* at an early time point after immunization, we detected no IL-1β in either lavages or supernatants of macrophages isolated 6 hours after MVAΔIL-1βR immunization (data not shown), indicating a certain resistance of primary macrophages to produce IL-1β upon MVA stimulation. This suggests that primary macrophages only respond to MVA in an activated state, at least in the C57BL/6 background.

### MVAΔIL-1βR infection increases amounts of bioactive IL-1β in human monocytic cells

**Figure 4:**
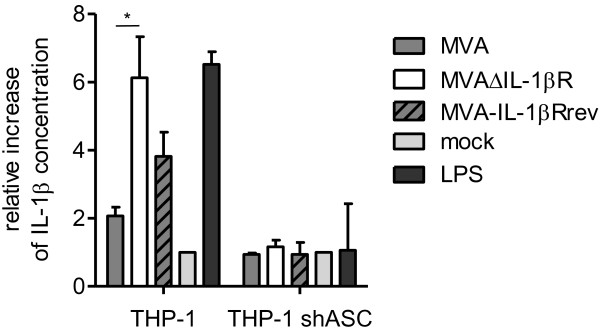
**MVAΔIL-1βR increases IL-1β biological activity after infection of human monocytic cells.** THP-1 or THP-1 shASC (down-regulated inflammasome) cells were pre-stimulated with 7.8 ng/ml PMA for 8 hours and subsequently infected with the indicated viruses at five MOI. 16 hours after infection, cell-free supernatants were transferred onto HEK-Blue IL-1β reporter cells, which respond to human IL-1β by expressing a reporter gene (SEAP). HEK-Blue IL-1β reporter cells were then incubated overnight and induced SEAP levels were measured by spectrophotometry. The intensity of the color reaction is proportional to the amount of IL-1β transferred from the culture supernatant of the infected THP-1 cells. THP-1 cells treated with 1 μg/ml LPS served as a positive control. IL-1β activity transferred from infected THP-1 cells is given relative to the amount of IL-1β produced in uninfected THP-1 cells (=1). Means of two independent infections per cell type and virus are shown with standard deviations. Data were analyzed with an unpaired *t*-test using Graph Pad Prism software. (* = p < 0.05).

To examine the biological activity of MVA-mediated IL-1β in the presence or absence of vIL-1βR in human cells, we infected monocytic THP-1 cells with MVAwt, MVAΔIL-1βR, or MVA-IL-1βRrev. The biological activity of induced IL-1β was determined by transferring cell culture supernatants onto HEK-293 reporter cells that specifically and quantitatively respond to exogenously applied free IL-1β [20]. Supernatants from THP-1 cells infected with MVAΔIL-1βR stimulated the highest levels of reporter gene expression, demonstrating that more biologically active free IL-1β was available than in other infections or THP-1 mock controls (Figure 4, left block). These data demonstrate that vIL-1βR reduces the biological activity of IL-1β produced in response to MVA infection in human cells.

We also analyzed the inflammasome contribution in THP-1 cells where expression of the adaptor molecule apoptosis-associated speck-like protein (ASC), a component of many inflammasomes [16], was down-regulated by shRNA [21]. Upon infection with the MVA derivates, even MVAΔIL-1βR was unable to stimulate IL-1β activity, as reflected by negligible reporter cell responses (Figure 4, right block). This confirms previous studies [8] demonstrating the impact of the inflammasome on MVA-mediated IL-1β induction in human cells.

## Conclusion

MVA can induce IL-1β in different antigen presenting cells, including murine myeloid dendritic cells. Whereas in these cells MyD88 and Trif are clearly required for IL-1β production, caspase-1 only seems to be involved in some cases. Moreover, only when the viral IL-1β receptor is absent can MVA infection stimulate high levels of free IL-1β to accomplish the cytokine´s biological function. Thus, deleting the gene encoding the viral IL-1β receptor offers a viable strategy for increasing inflammatory responses in order to enhance MVA-based vaccine immunogenicity.

## Material and methods

### Mice, cells and viruses

Female C57BL/6 mice (Charles River Laboratories, Sulzfeld, Germany), MyD88^−/−^TRIF^−/−^ mice (C57BL/6 background) [5,22] and Caspase-1^−/−^ mice [23] were bred and treated as previously described [5]. Mouse experimental work was approved by the State Government of Hessen, Germany (authorization no. F107-103). All animals were handled in compliance with German Animal Welfare Regulations.

The following cells were cultivated as described in each reference: primary chicken embryo fibroblast (CEF) [24], HEK-Blue IL-1β reporter cells (Cayla-InvivoGen, Toulouse, France) [20], monocytic THP-1 (DSMZ, Braunschweig, Germany) and THP-1 shASC [21] (both cultivated according [6]); primary mouse cells [5], additionally supplemented with 1 mM sodium pyruvate, 1 mM HEPES, 0.1 mM 2-mercaptoethanol.

MVA [2,4] and recombinant MVA [12] were propagated and titrated in CEF as described [24].

### Cell isolation from mice and *in vitro* differentiation

Bone marrow derived macrophages were generated as described [5]. Myeloid dendritic cells were isolated and generated accordingly using GM-CSF for differentiation (100 ng/ml, Peprotech GmbH, Hamburg, Germany) and 8-day cultivation. Cells from the peritoneal cavity were isolated from sacrificed mice by flushing out cells through injection of 5–7 ml of PBS into the abdominal cavity.

### Infection of cells

Cells were infected with indicated viruses at a multiplicity of infection (MOI) of five, washed after one hour, and cultivated as described above. As positive controls, cells were treated with 1 μg/ml LPS (from *Salmonella typhimurium*, Sigma-Aldrich, Steinheim, Germany). Harvested supernatants were cleared from cells and debris by centrifugation at 1200 × g.

### Quantification of IL-1β by ELISA

Cell-free supernatants were diluted with 1 volume PBS/10% FCS and subjected to IL-1β ELISA (BD Biosciences, Heidelberg) performed according to the manufacturer´s instructions.

### IL-1β bioactivity assay

PMA-stimulated THP-1 or THP-1 shASC cells were infected with the indicated viruses and supernatants transferred 16 hours after infection onto HEK-Blue™ IL-1β cells (Cayla-Invivogen, Toulouse, France). Treatment of HEK-Blue cells and colorimetric assay was performed according to the manufacturer´s instructions [20].

### FACS analysis of spleen cells and macrophages

Spleen cells of sacrificed mice were stimulated with 1 μg/ml VACV-specific peptides B8R_20-27_ or K3L_6-15_ or as a control, β-galactosidase_876_ peptide (Thermo Electron Corp. Ulm, Germany) [15] for two hours, then another three hours after adding 1 μl of Golgi Plug (Becton Dickinson, Heidelberg, Germany). For intracellular cytokine stain, cells were treated with anti-CD16/CD32-Fc-block, followed by antibodies directed against CD8 and CD62L. Cells were fixed and permeabilized using Cytofix/Cytoperm according to the manufacturer´s instructions (BD Biosciences, Heidelberg, Germany) followed by staining for INF-γ. Each incubation step was performed for 20 min at 4°C. Cells were finally fixed with 2.5% formaldehyde and analyzed by FACS. Macrophages were blocked as described for spleen cells and then stained for activation markers CD80 or MHCII in FACS buffer (PBS/2% BSA/20 mM EDTA/0.03% sodium azide) followed by staining for F4/80, CD11b and CD11c. All steps were performed for 30 min interspersed by washing rounds with FACS buffer. Cells were fixed with 1% paraformaldehyde and analyzed by FACS.

Source of FACS staining antibodies: MHCII (PE), CD11b (PerCP-Cy5.5), CD11c (APC), CD8 (PacBlue), CD62L (APC), INF-γ (PE) and anti-CD16/CD32-Fc-block are from BD Biosciences (Heidelberg, Germany), CD80 (FITC) from eBioscience (Frankfurt/Main, Germany) and F4/80 (PacBlue) from AbD Serotech (Düsseldorf, Germany). Data acquired by FACS analysis on a LSRII using FACSDiVa software (BD Biosciences) were analyzed with FCS express (De Novo Software; Figure 3B) or FlowJo (Tree Star Inc.; Figure 1).

## Abbreviations

BMDM: Bone marrow-derived macrophages; IFN: Interferon; IL-1β: Interleukin-1beta; mDC: Myeloid dendritic cells; MOI: Multiplicity of infection; MVA: Modified vaccinia virus Ankara; MVAwt: MVA wild-type; MyD88: Myeloid differentiation primary response gene 88; PEC: Peritoneal exudate cells; SEAP: Secreted alkaline phosphatase; TLR: Toll-like receptor; VACV: Vaccinia virus; vIL-1βR: Viral interleukin-1beta receptor; wt: Wild-type.

## Competing interests

GS is an inventor and has a patent application concerning the mutant virus MVAΔIL-1βR and its use in immunotherapy and vaccination, in particular in the prevention and therapy of cancer and infectious diseases. The other author(s) declare that they have no competing interests.

## Authors’ contributions

SZ, TR and AS performed experiments; SZ, AS, ZW, GS conceived and designed experiments, and analyzed and interpreted the results; AS drafted and SZ, TR, ZW and GS revised the manuscript. All authors read and approved the final manuscript.
